# Combination of CNP, MT and FLI during IVM Significantly Improved the Quality and Development Abilities of Bovine Oocytes and IVF-Derived Embryos

**DOI:** 10.3390/antiox12040897

**Published:** 2023-04-07

**Authors:** Peipei Zhang, Baigao Yang, Xi Xu, Hang Zhang, Xiaoyi Feng, Haisheng Hao, Weihua Du, Huabin Zhu, Shujing Li, Wenli Yu, Adnan Khan, Saqib Umer, Xueming Zhao

**Affiliations:** 1 Institute of Animal Sciences (IAS), Chinese Academy of Agricultural Sciences (CAAS), No. 2 Yuanmingyuan Western Road, Haidian District, Beijing 100193, China; 2 Shijiazhuang Tianquan Elite Dairy Ltd., Shijiazhuang 050200, China; 3 Agricultural Genomics Institute at Shenzhen, Chinese Academy of Agricultural Sciences, Shenzhen 518120, China; 4 Department of Theriogenology, University of Agriculture, Faisalabad 38000, Punjab, Pakistan

**Keywords:** bovine, oocytes, ovum pich up, C-type natriuretic peptide, melatonin, FLI (IGF+FGF+LIF)

## Abstract

Oocyte maturation is a critical step in the completion of female gametogenesis in the ovary; thus, for subsequent fertilization and embryogenesis. Vitrification of embryo also has been shown to be closely associated with oocyte maturation. To improve the quality and developmental potential of bovine oocytes derived from in vitro maturation (IVM), Pre-IVM with C-type natriuretic peptide (CNP), melatonin (MT) and in combination, IGF1, FGF2, LIF (FLI) were supplemented in the IVM medium. In this current study, we cultured bovine oocytes in Pre-IVM with CNP for 6 h before transferring them to the IVM medium supplemented with MT and FLI. The developmental potential of bovine oocytes was then investigated by measuring the reactive oxygen species (ROS), the intracellular glutathione (GSH) and ATP levels, the transzonal projections (TZP), the mitochondrial membrane potential (ΔΨm), cacline-AM, and the expression of related genes (cumulus cells (CCs), oocytes, blastocysts). The results revealed that oocytes treated with a combination of CNP, MT, and FLI had dramatically improved the percentage of oocytes developed to blastocyst, ATP content, GSH levels, TZP intensity, the ΔΨm, cacline-AM fluorescence intensity, and considerably reduced ROS levels of oocytes. Furthermore, the survival rate and the hatched rate after vitrification of the CNP+MT+FLI group were significantly higher than those other groups. Thus, we speculated that CNP+MT+FLI increases the IVM of bovine oocytes. In conclusion, our findings deepen our understanding and provide new perspectives on targeting the combination of CNP, MT and FLI to enhance the quality and developmental potential of bovine oocytes.

## 1. Introduction

The breeding industry strongly supports the optimization of ovum pick up (OPU) and in vitro embryo production (IVP) as a way to improve genetics in dairy and beef cattle [1]. OPU combined with in vitro fertilization (IVF) is widely used for the genetic improvement of bovine embryos [2]. The embryo production efficiency of OPU-IVF has been reported to be higher than in vivo embryo production [3]. In order to maximize the breeding potential of elite cattle, OPU-IVF can hasten the breeding process by producing a large number of offspring from cattle with high genetic value; hence, increasing the selection intensity and decreasing the generation interval [4].

Even though there have been significant advancements in OPU technology, the production rate of in vitro blastocysts is still low [5]. The main reason for this reduced rate is that the developmental competence of OPU oocytes obtained through in vitro maturation (IVM) is lower compared to oocytes matured in vivo [6]. Synchronization of oocyte meiosis and cytoplasmic maturation is important for successful fertilization and for development before and after implantation [7], although it remains a challenge during IVM of oocytes. Oxidative stress is considered to be one of the factors of the impaired developmental capacity of oocytes and embryos [8]. Hence, enhancing the culture condition is crucial for boosting the quality and the developmental competence of oocytes derived from OPU.

Recent research have shown that C-type natriuretic peptide (CNP) can improve cytoplasmic maturation by maintaining sufficient cAMP levels to sustain the meiotic arrest of bovine oocytes for 6–8 h [9]. melatonin (MT) is a neurohormone secreted by the pineal gland in vertebrates and partly by other peripheral organs such as the retina, the intestine, immune-competent cells, and the gonads [10]. As a potent antioxidant, MT has been utilized to reduce intracellular oxidative stress and to prevent DNA damage in mouse oocytes [11], and decrease the generation of reactive oxygen species (ROS) during the maturation of mouse [12] and bovine [13] oocytes. Growth factors such as Insulin-like growth factor-1 (IGF1) can promote the rate and quality of oocyte maturation; subsequently, improving embryo development [14,15]. Similarly, fibroblast growth factor 2 (FGF2) can enhance nuclear maturation, cumulus cells (CCs)’ survival, and the extracellular matrix quality of bovine oocytes [16]. The leukemia inhibitory factor (LIF) promoted oocyte nuclear maturation and blastocyst development in porcine [17] and bovine [18] oocytes. Importantly, the supplementation of IGF1, FGF2, and LIF (FLI medium) improved porcine [19,20,21] and sheep [22] embryonic development in vitro. However, the mechanism through which the combination of CNP, MT, and FLI enhances the maturation and developmental capacity of oocytes in vitro has not been fully elucidated.

Therefore, the aim of this present study was to investigate how a Pre-IVM system with CNP, and supplementation with MT and FLI during IVM, affected the maturation quality and development potential of bovine oocytes. The findings of this study will contribute to establishing an efficient approach to improve the maturation and developmental capacity of bovine oocytes.

## 2. Materials and Methods

Animals were treated according to the recommendations of the Institutional Animal Care and Use Committee of the Chinese Academy of Agricultural Sciences.

### 2.1. Oocyte Collection

During the IVM experiment, an average of 5–8 oocytes were obtained per ovary, and the procedure was performed on 40 ovaries per session. Bovine ovaries were collected from the slaughterhouse and transported to the laboratory within 2 h at a temperature of 30–35 °C. Cumulus-oocyte complexes (COCs) were extracted from 2–8 mm follicles using a sterile 18-gauge needle. COCs were washed and those with at least 3 layers of CCs were chosen to be use for the experiment.

For the OPU experiment, an average of 20 oocytes were extracted per donor, and 4 donors were worked on per session. Oocytes were collected through an ultrasound-guided transvaginal procedure. Before OPU, donors were not given any hormone treatments. All collected COCs from a given donor and OPU session were treated together as one batch during the whole in vitro production procedure.

### 2.2. Oocytes Pre-IVM and IVM

The oocytes in the experiment were cultured in different IVM media as per the experiment design. The control group involved culturing the COCs in 4-well plates, with each well containing 500 µL of a basic IVM medium made up of TCM199 (Gibco BRL, Carlsbad, CA, USA) supplemented with 10% fetal bovine serum (FBS, Hyclone; Gibco BRL), 1 µg/mL estradiol, 10 µg/mL follicle-stimulating hormone (FSH), 50 ng/mL epidermal growth factor, and 10 µg/mL lutrinizing hormone (LH) for 22–24 h at a temperature of 38.5 °C under 5% CO_2_. In the first experiment, the COCs were first cultured in a pre-IVM medium containing TCM199 supplemented with 10% FBS and CNP (100 nM) in 4-well plates for 6 h at 38.5 °C under 5% CO_2_. They were then transferred to the basic IVM medium for an additional 22–24 h under the same conditions. For the second experiment, the COCs were cultured in a pre-IVM medium with CNP for 6 h at 38.5 °C under 5% CO_2_; then, transferred to the basic IVM medium with 10^−9^ M MT for 22–24 h under the same conditions. In the third experiment, the COCs were first cultured in a pre-IVM medium with CNP for 6 h at 38.5 °C under 5% CO_2_; then, transferred to the basic IVM medium with MT and FLI (IGF1 (20 ng/mL), FGF2 (40 ng/mL), LIF (20 ng/mL)) for 22–24 h under the same conditions.

### 2.3. IVF of Oocytes

The IVF procedures were carried out based on the techniques outlined by Brackett et al. [23] with some slight modifications. A straw of frozen semen was thawed in a water bath at 38 °C and mixed with 7 mL of washing medium in a 15 mL centrifuge tube. The mixture was then centrifuged twice at 1500 r/min for 5 min each time. The supernatant was discarded and the semen concentration was diluted to 1 × 10^7^/mL; then, 10 μL of the sperm suspension was mixed with 90 μL of the fertilization medium, which contained 20–30 oocytes for insemination. After 16–18 h, zygotes were transferred to CR1aa medium for 48 h; then, the cleaved embryos were transferred to CR1aa medium containing 10% FBS for 5 days, and half of the medium was replaced every 48 h.

### 2.4. Vitrification and Thawing of Blastocyst

The methods of vitrification and thawing were changed slightly with reference to Zhao et al. [24]. For vitrification, blastocysts were exposed to a mixture of 10% EG and 10% DMSO for 30 s; then, placed in EDFSF40 for 25 s. The EDFSF40 consisted of FSF solution with 20% (*v*/*v*) EG and 20% (*v*/*v*) DMSO. The FSF solution contained 300 g/L Ficoll, 0.5 M sucrose, and 20% (*v*/*v*) FBS in the DPBS medium. Afterward, the blastocysts were immediately immersed in liquid nitrogen after being absorbed by an OPS. For thawing, the OPS was taken out of the liquid nitrogen and placed in 0.25 M sucrose solution, incubated for 1 min, followed by transfer to a 0.15 M sucrose solution at 38.5 °C for 5 min.

### 2.5. Analysis of ROS, ATP, and GSH in Oocytes

The ROS levels of the oocytes were evaluated according to Rahimi et al. [25] with some modifications. A group of 10 oocytes was washed 3 times with washing solution (0.1% polyvinyl alcohol (PVA)) and transferred to an M199 medium with fluorescent dye 2′,7-dichlorodihydrofluorescein diacetate (H2DCF-DA; Genmed Scientific Inc., Wilmington, DE, USA). Ten oocytes were incubated at 37 °C for 20 min; then, they were washed 3 times with washing solution and transferred into 96-well dishes. Luminescence was measured by a luminometer (Infinite M200; Tecan Group Ltd., Männedorf, Switzerland) and the abundance of ROS was expressed as photon counts per s (cps).

The ATP levels of a group of 10 oocytes were assessed using the ATP Bioluminescence Assay Kit (ATP Bioluminescence Assay Kit HS II, Roche diagnostics, GmbH Mannheim, Germany), as described in a study by Zhao et al. [26]. Ten oocytes were lysed using 20 μL of ATP-releasing agent. Samples and standard solutions were added to 96-well dishes plates, and each well contained 100 μL ATP detection solution. Luminescence was immediately measured by luminometer (InfiniteM200, Tecan Group Ltd) for 10 s. ATP levels of the samples were calculated based on a standard curve established using internal standards.

The glutathione (GSH) levels of oocytes were assessed using the procedure outlined by Ozawa et al. [27]. A group of 10 oocytes was washed 3 times in Dulbecco’s phosphate buffer solution (DPBS) containing 1 mg/mL polyvinylpyrrolidone (PVP); then, transferred into a microtube containing 50 µL of 6 mM DTNB, 350 µL of 0.33 mg/mL nicotinamide adenine dinucleotide phosphate (NADPH), and 90 µL of distilled water. To start the reaction, 5 μL of 250 U/mL GSH reductase was added. Absorbance was monitored at 412 nm with a spectrophotometer for 3 min, with readings recorded every 30 s. The total GSH content in the oocytes was calculated according to the constructed standard curve.

### 2.6. Analysis of TZPs in Oocytes

The transzonal projections (TZP) immunofluorescence staining in the oocytes was assessed using the procedure outlined by Yuan et al. [19]. The oocytes were washed 3 times in PBS containing 0.1% PVA, fixed with paraformaldehyde at 37 °C for 30 min, treated with 0.1% Triton X-100 for 5 min, and blocked with a 1 mg/mL BSA-PVA-PBS solution for 1 h. They were then incubated in Rhodamine Phalloidin (ab235138, 1:1000 dilution; Abcam, Cambridge, UK) for 2 h at 37 °C in the dark; then, imaged with confocal microscopy (TCS SP8; Lecia). The fluorescent intensity of the TZP staining was analyzed with ImageJ-Pro-Plus 6.0 software (Version1.40; National Institutes of Health). The fluorescent pixel values of the oocytes were measured from 10 different zona pellucida regions, and each region had the same area; then, the background fluorescence values were subtracted from the final values, and the statistically significant differences among the groups were analyzed.

### 2.7. qRT-PCR of Candidate Genes in CCs, Oocytes and Blastocysts

COCs were denuded of CCs by treatment with 0.01% hyaluronidase. The total RNA from CCs and oocytes was extracted using TRIzol reagent (Invitrogen, Waltham, MA, USA) as per the manufacturer’s instructions. The total RNA was reversely transcribed to synthesize the first-strand cDNA using random hexamers. The gene’s expression levels of blastocysts was measured using the Single Cell-to-CT quantitative real-time PCR kit (Life Technologies, Carlsbad, CA, USA), following the manufacturer’s protocol. qRT-PCR of mRNA was performed using an ABI 7500 SDS instrument (Applied Biosystems, Foster City, CA, USA) using the following parameters: a 2 min preheat at 95 °C, followed by 40 cycles of 10 s at 95 °C and 30 s at 60 °C. The gene expression fold change was analyzed with the 2^−△△Ct^ method, with β-actin used as the reference gene and the results expressed as a ratio to β-actin levels. The PCR primers used in this study are shown in Table 1.

### 2.8. ΔΨm Examination of Oocytes

The mitochondrial membrane potential (ΔΨm) of oocytes was evaluated by utilizing JC-1 staining (Molecular Probes, Sigma-Aldrich, Missouri, MO, USA). In brief, the oocytes were gathered and incubated with 10 μg/mL JC-1 in 5% CO_2_ at 38.5 °C for 30 min. Afterward, they were rinsed 3 times with DPBS and subjected to analysis using a laser-scanning confocal microscope (TE2000-U; Nikon, Tokyo, Japan). The examination was conducted using a 488 nm wavelength, with the emission wavelength set at 530 nm. The fluorescent pixel values of the oocytes were measured from 10 different cytoplasmic regions and 10 different cortical regions, and each region had the same area; then, the cytoplasmic mean was subtracted from the cortical mean. The red and green fluorescence ratio was used to analyze ΔΨm.

### 2.9. Calcein-AM Staining of Oocytes

After IVM, the oocytes were mechanically denuded and transferred to 1 μM calcein-AM labeled for 25 min. The oocytes were washed 3 times in DPBS and then immediately examined by the fluorescence microscopy (Nikon, Tokyo, Japan). The fluorescence intensity of calcein-AM is in pixels [28]. The fluorescent pixel values of the oocytes were measured from 10 different cytoplasmic regions, and each region had the same area; then, the background fluorescence values were subtracted from the final values, and the statistically significant differences among the groups were analyzed.

### 2.10. Statistical Analysis

The data were presented as mean ± standard error. The analysis was performed using a one-way analysis of variance (anova) with Duncan’s test through SAS software (SAS Institute, Cary, NC, USA), and *p* < 0.05 was deemed statistically significant. All the experiments were repeated at least 3 times.

## 3. Results

### 3.1. Effect of the Combination Treatment of CNP, MT, and FLI on the Maturation of Bovine Oocytes Collected Post Mortem or Intra Vitam by OPU Method

As shown in Table 2, the nuclear maturation rate of the bovine oocytes collected post mortem of the CNP+MT+FLI group (92.95 ± 2.48%) was significantly higher than that of the CNP group (83.69 ± 4.85%), the CNP+MT group (85.33 ± 3.57%), and the control group (75.61 ± 5.02%; *p* < 0.05). Furthermore, the nuclear maturation rate of the oocytes collected Intra Vitam by OPU of the CNP+MT+FLI group (90.48 ± 3.14%) was significantly higher than that of the control group (81.66 ± 2.63; *p* < 0.05).

### 3.2. Effect of the Combination Treatment of CNP, MT, and FLI on the Developmental Ability of Bovine Oocytes and IVF-Derived

The cleavage rate and the blastocysts development rate of the CNP+MT+FLI group (91.61 ± 5.17%, 51.41 ± 4.73%) were significantly higher than those of the CNP group (79.58 ± 3.14%, 35.40 ± 1.90%), the CNP+MT group (85.03 ± 4.84%, 42.40 ± 3.94%), and the control group (73.51 ± 2.47%, 30.15 ± 2.15%; *p* < 0.05). The survival rate and the hatched rate after vitrification of the CNP+MT+FLI group (97.37 ± 5.35%, 94.59 ± 8.23%) were significantly higher than those of the CNP group (90.00 ± 8.43%, 85.19 ± 7.26%), the CNP+MT group (91.43 ± 7.19%, 87.50 ± 6.48%), and the control group (90.32 ± 8.17%, 80.77 ± 6.75%; *p* < 0.05). For the OPU oocytes, the cleavage rate and the blastocysts development rate of the CNP+MT+FLI group (89.36 ± 4.25%, 41.67 ± 2.17%) were significantly higher than those of the control group (75.22 ± 4.38%, 30.49 ± 1.87%; *p* < 0.05), as presented in Table 3.

### 3.3. Effect of the Combination Treatment of CNP, MT, and FLI on Gene Expression in Bovine IVF Blastocysts

**Figure 1:**
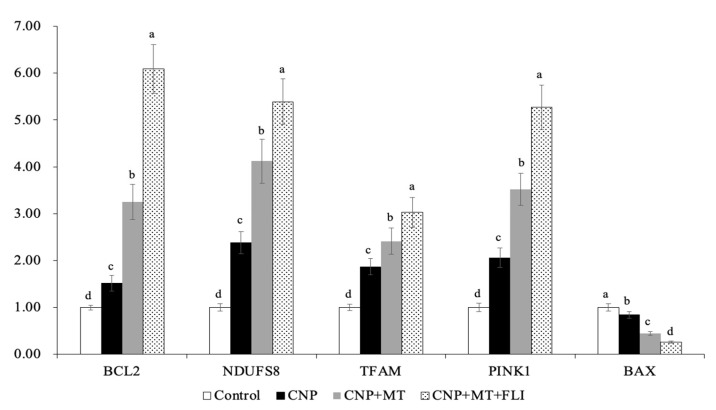
Effect of the combination treatment of CNP, MT, and FLI on the gene expression in blastocysts. a, b, c, d Values with different superscripts indicate significant differences between groups (*p* < 0.05).

Meanwhile, as shown in Figure 1, the mRNA expression levels of mitochondrial genes (*NDUFS8*, *TFAM*, *PINK1*) in the blastocysts of the CNP+MT+FLI group were significantly higher compared to the other groups. The mRNA expression of the anti-apoptosis gene *BCL2* in the CNP+MT+FLI group was significantly higher compared to the other groups, which was in contrast to the expression of the pro-apoptosis gene *BAX* (*p* < 0.05).

### 3.4. Effect of the Combination Treatment of CNP, MT, and FLI on ROS, ATP, and GSH Levels in Bovine Oocytes

**Figure 2:**
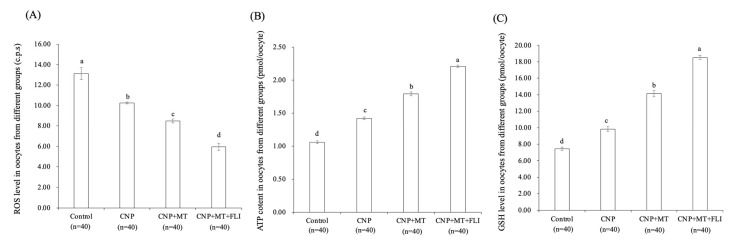
Effect of the combination treatment CNP, MT, and FLI on the ROS, ATP, and GSH levels in bovine oocytes. (**A**) Effect of CNP, MT, and FLI on ROS level in bovine oocytes. (**B**) Effect of CNP, MT, and FLI on ATP content in bovine oocytes. (**C**) Effect of CNP, MT, and FLI on GSH level in bovine oocytes. a, b, c, d Values with different superscripts indicate significant differences between groups (*p* < 0.05).

The ROS levels in the oocytes of the CNP group, the CNP+MT group, and the CNP+MT+FLI group were significantly lower compared to the control group, and the ROS level of the CNP+MT+FLI group was significantly lower compared to the other groups (Figure 2A). The ATP and GSH levels in the oocytes of the CNP group (1.43 ± 0.03 pmol, 9.67 ± 0.04 pmol), the CNP+MT group (1.76 ± 0.02 pmol, 13.99 ± 0.27 pmol), and the CNP+MT+FLI group (2.23 ± 0.06 pmol, 18.40 ± 0.17 pmol) were significantly higher (*p* < 0.05) compared to the control group (1.05 ± 0.02 pmol, 8.09 ± 0.87 pmol), and the ATP and GSH levels of the CNP+MT+FLI group were significantly higher (*p* < 0.05) compared to the other groups (Figure 2B,C).

### 3.5. Effect of the Combination Treatment of CNP, MT, and FLI on TZPs in Bovine Oocytes

**Figure 3:**
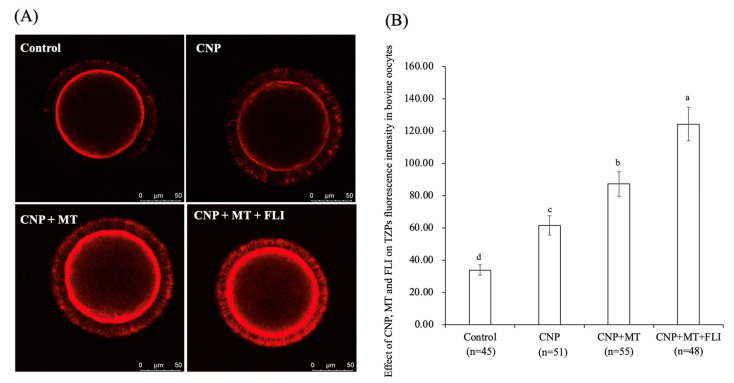
Effect of the combination treatment CNP, MT, and FLI on TZPs fluorescence intensity in bovine oocytes. (**A**) Representative image of TZPs staining in bovine oocytes. (**B**) Effect of the combination treatment of CNP + MT + FLI on TZPs in bovine oocytes. Bar = 50 μm. a, b, c, d Values with different superscripts indicate significant differences between groups (*p* < 0.05).

As shown in Figure 3, the fluorescence intensity of the CNP group, the CNP+MT group, and the CNP+MT+FLI group was significantly higher compared to the control group, and the fluorescence intensity of the CNP+MT+FLI group was significantly lower compared to the other groups (*p* < 0.05).

### 3.6. Effect of the Combination Treatment of CNP, MT, and FLI on the ΔΨm in Bovine Oocytes

**Figure 4:**
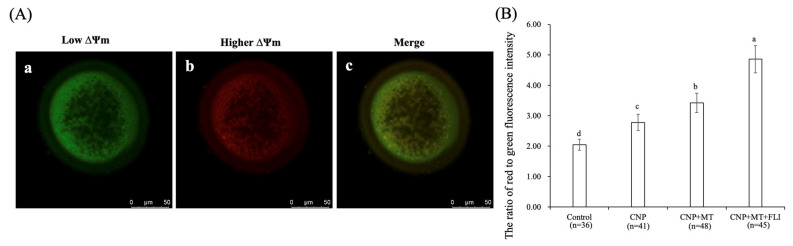
Effect of the combination treatment CNP, MT, and FLI on the ΔΨm in bovine oocytes. (**A**) Representative image staining of JC-1 staining in bovine oocytes. a: Green: JC-1 monomeric form (low ΔΨm); b: Red: JC-1-aggregated form (higher ΔΨm); c: merging of images with green and red fluorescence. Bar = 20 μm. (**B**) Effect of CNP+MT+FLI on the ratio of red to green fluorescence intensity in bovine oocytes. a, b, c, d Values with different superscripts indicate significant differences between groups (*p* < 0.05).

As shown in Figure 4, the ΔΨm of the CNP group, the CNP+MT group, and the CNP+MT+FLI group was significantly higher compared to the control group, and the ΔΨm of the CNP+MT+FLI group was significantly higher compared to the other groups (*p* < 0.05).

### 3.7. Effect of the Combination Treatment of CNP, MT, and FLI on the Calcein-AM Fluorescence Intensity of Bovine Oocytes

**Figure 5:**
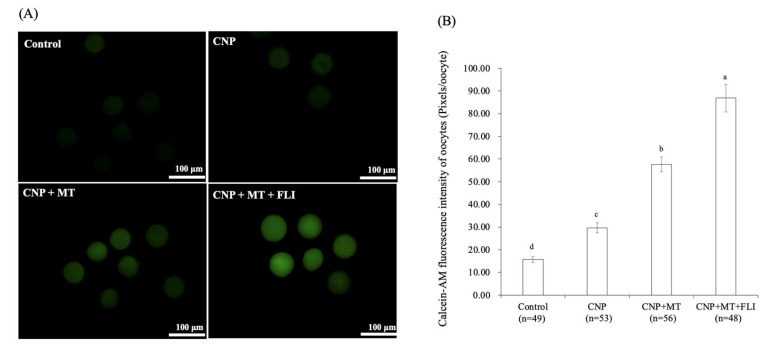
Effect of the combination treatment CNP, MT, and FLI on calcein-AM fluorescence intensity of bovine oocytes. (**A**) Representative image staining of calcein-AM in bovine oocytes. Bar = 100 μm. (**B**) Effect of CNP+MT+FLI on the calcein-AM fluorescence intensity of bovine oocytes. a, b, c, d Values with different superscripts indicate significant differences between groups (*p* < 0.05).

As shown in Figure 5, the calcein-AM fluorescence intensity of the CNP group, the CNP+MT group, and the CNP+MT+FLI group was significantly higher compared to the control group, and the calcein-AM fluorescence intensity of the CNP+MT+FLI group was significantly higher compared to the other groups (*p* < 0.05).

### 3.8. Effect of the Combination Treatment of CNP, MT, and FLI on Gene Expression in Bovine Oocytes and CCs

**Figure 6:**
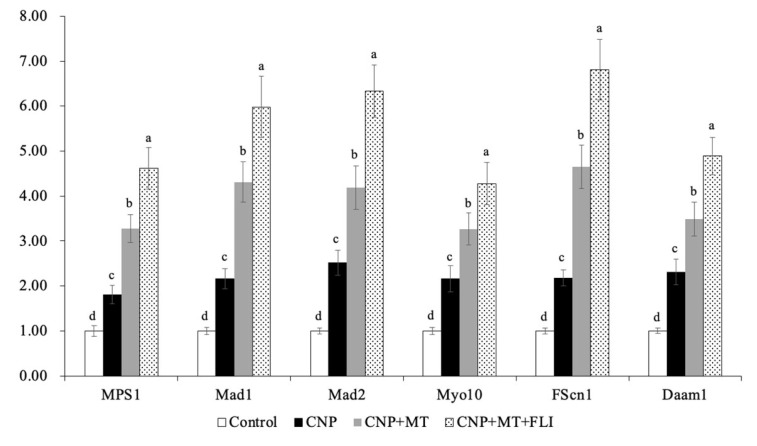
Effect of the combination treatment CNP, MT, and FLI on the gene expression in bovine oocytes. a, b, c, d Values with different superscripts indicate significant differences between groups (*p* < 0.05).

**Figure 7:**
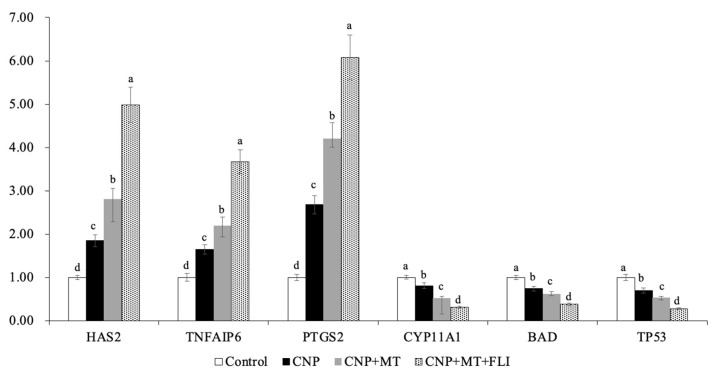
Effect of the combination treatment of CNP, MT, and FLI on the gene expression in CCs. a, b, c, d Values with different superscripts indicate significant differences between groups (*p* < 0.05).

Meanwhile, as shown in Figure 6, the mRNA expression levels of the spindle gene (*MPS1*), the mitotic arrest deficient genes (*Mad1*, *Mad2*), and the TZP-related genes (*Myol0*, *FScn1*, *Daam1*) in the oocytes of the CNP+MT+FLI group were significantly higher compared to the other groups (*p* < 0.05). The mRNA expression levels of the cumulus expansion-related genes (*HAS2*, *TNFAIP6*, *PTGS2*) in CCs of the CNP+MT+FLI group were significantly higher compared to the other groups, and the stress-related genes (*CYP11A1*, *BAD*, *TP53*) in CCs of the CNP+MT+FLI group were significantly lower compared to the other groups (*p* < 0.05), as shown in Figure 7.

## 4. Discussion

Recent studies revealed that pre-IVM with CNP enhanced the developmental competence of the bovine oocytes [29,30,31]. Our study also demonstrated that the use of a novel IVM system based on CNP pretreatment considerably enhanced the developmental competence of the bovine oocytes (Table 2), which was due to CNP increased oocytes cGMP levels [32]. Evidence from numerous studies on humans [33], bovine [26,34,35], and pigs [36,37] has shown that MT supplementation was beneficial for the IVM of oocytes and their development; and, reducing ROS levels depicts why the combination treatment of CNP and MT significantly enhanced the developmental competence of the bovine oocytes. The oocytes treated with CNP, MT, and FLI showed the highest development potential because FLI promoted porcine oocyte IVM by activating MAPK1/3 [19,38].

Earlier studies have found that CNP may raise cGMP levels, which would boost the maturation rate and the development of vitrified mouse oocytes [39], explaining the survival and hatching rate of the CNP group following vitrification. MT improves the development ability of the vitrified oocytes by reducing ROS [24], and the addition of FLI to the maturation medium of the bovine oocytes can increase cytoskeleton integrity and decrease post-thaw cell apoptosis, and improve the developmental ability of the vitrified bovine oocytes [40]. Our finding explained that the survival and hatching rates of the CNP+MT+FLI group after vitrification were significantly greater than those of the control group, as indicated in Table 3.

*TFAM* is an autosomal mitochondrial gene that is essential for the transcription and replication of mitochondrial DNA, and silencing *TFAM* induces mitochondrial dysfunction [41]. In addition, *PINK1* is stabilized on the outer membrane of depolarized mitochondria to initiate mitophagy [42]. *NDUFS8* is a subunit of the NADH dehydrogenase (ubiquitin) complex; it is located in the inner mitochondrial membrane and is involved in the electron transport chain [43]. The results of our research revealed that the expression levels of *TFAM*, *PINK1*, and *NDUFA8* were significantly increased due to CNP+MT+FLI, indicating an improvement in mitochondrial function (Figure 1). Moreover, BAX is a pro-apoptotic protein that causes cell death, while BCL-2 is an anti-apoptotic protein that promotes cell survival [44]. Our results demonstrated that the apoptosis of blastocyst was suppressed by the combination treatment of the CNP+MT+FLI because the expression level of the *BCL-2* gene was significantly elevated, and the expression level of the *BAX* gene was decreased (Figure 1).

Foregoing research have found that pre-IVM CNP treatment can significantly reduce ROS levels by inducing SIRT1 abundance and the regulation of genes involved in antioxidant defense [31], promoting ATP production by stimulating mitochondrial development in oocytes [45,46]. GSH was transferred from bovine CCs to oocytes via gap junctions and extended communication functions, which contributed to the accumulation of GSH in the oocytes [30]. These shreds of evidence help to explain the increased ATP and GSH levels, and the decreased ROS levels in the CNP group (Figure 2). In oocytes, MT promotes the antioxidant defense system (NADPH and GSH) and the capacity to scavenge ROS by increasing G6PDH activity [47]. Meanwhile, MT also increases mitochondrial energy production by activating the SIRT1/PGC-1α pathway [48]. In bovine and porcine oocytes, MT supplementation has been shown to decrease intra-oocyte H_2_O_2_ and boost intra-oocyte GSH and ATP levels [26,49]. Previous experiments showed that the treatment with IGF1 significantly increased the GSH and decreased the ROS level in oocytes [50]. In addition, IGF1 may act by modulating the expression of the cytochrome c oxidase subunit 1 gene, which, in turn, increases ATP levels by acting indirectly on the respiratory chain activity of oocytes [51]. Hence, we found the highest level of GSH and ATP, and the lowest level of ROS in the CNP+MT+FLI group.

During the development of follicles and the growth of oocytes, TZPs emerge from CCs and penetrate the zona pellucida. Some TZPs reach the oolemma to establish a direct link between the oocytes and granulosa cells [52]. Communication through TZPs and gap junctions is crucial for the regulation of meiosis in oocytes and the maturation of the oocyte [53,54]. CNP increases cAMP levels by regulating cGMP, and cAMP is mediated by TZPs from CCs to the oocyte [55]. It has been proven that CNP-treated oocytes showed a higher density of TZP and functionally open gap junctions [55,56]. Similarly, our experiments also showed that the TZP level in the oocytes of the CNP group was significantly higher than that of the control group. Meanwhile, supplementation of MT increased the number and density of TZPs by improving the gap junction intercellular communication (GJIC) activity of bovine oocytes [28]. It was shown that FLI increased oocyte TZPs through activating MAPK activation [19]. All of these studies supported the highest TZP level in the CNP+MT+FLI group. Oocyte maturation and development are supported by CCs transferring metabolites and signaling molecules to the oocytes [57], indicating that the development ability of the CNP+MT+FLI group was improved.

Mitochondria are the major source of ATP, and play a vital role in cellular metabolism; ΔΨm activity is closely related to both the developmental potential of oocytes and embryos [58]. It is reported that treatment with CNP before IVM increases the relative abundance of PGC-1α, NRF1, NRF2, and TFAM mRNA transcripts in bovine oocytes, leading to enhanced ΔΨm levels and mitochondrial function [31], which explains the higher ΔΨm level in the oocytes from the CNP group. MT also blocks MPTP to preserve the ΔΨm level of oocytes [59,60]. Furthermore, IGF-I may play a regulatory role in the increase of ΔΨm in oocytes by regulating the expression of the cytochrome c oxidase subunit 1 gene [51]. This evidence led to elucidating the rationale for the highest ΔΨm in the CNP+MT+FLI group (Figure 4B).

GJIC between the CCs and the oocytes was assessed using the fluorescent dye calcein after IVM. It was previously established that the increased level of calcein-AM fluorescence intensity in the oocytes of the CNP group was due to the increased gene expression of connexin alpha 1 and 4 (GJA1 and GJA4) in CCs and the ability of COCs in follicles to take up calcein through gap junctions [61]. Similarly, MT supplementation resulted in increased GJIC activity of bovine oocytes by expanding the GJA4 expression [28]. LIF stimulated the expression of GJA4 for GJIC during follicle development [62]. Our experiments showed that the CNP+MT+FLI group significantly improved the level of calcein-AM fluorescence intensity in oocytes, which explained the increased development ability of bovine oocytes (Figure 5).

*Myo10*, *FScn1* and *Daam1* are genes related to the key structural components of TZP in oocytes [47]; *Myo10* promotes the formation of actin-TZPs [63], *FScn1* mediates cell interactions and migration, and cytoplasmic microfilaments that contribute to cell structure and intracellular movements [64], and *Daam1* belongs to the formin family and regulates the nucleation of unbranched actin filaments [65]. A previous study revealed that MT significantly increased the mRNA levels of the *Myo10*, *Fscn1*, and *Daam1* in mice [47]. Our findings also showed that CNP+MT+FLI significantly increased the expression levels of *Myo10*, *FScn1*, and *Daam1*, which proved that the TZP in oocytes was enhanced (Figure 6). MPS1 is a bispecific kinase which is the core protein of SAC and regulates the mitotic recruitment of other SAC proteins [66]. SAC-related genes and proteins are believed to have a significant regulatory effect on the normal assembly of spindles and segregation of chromosomes during meiosis [67]. In particular, *Mad1*, *Mad2*, and *Mps1* are crucial in spindle assembly [68] and their proper expression is necessary for chromosome segregation and attachment to dynamic microtubules [67]. Notably, when MT was added to the IVM medium, there was a significant increase in the relative expression of *Mps1* and *Mad1* in developing mature oocytes [69]. Our study showed that the mRNA expression of *MPS1*, *Mad1*, and *Mad2* was significantly enhanced by the combination treatment of CNP, MT, and FLI, and this was because MT and growth factors can promote the development of oocytes (Figure 6).

HAS2 is the main enzyme for the synthesis of hyaluronic acid, which is necessary for the expansion of the CCs and signaling during ovulation [70]. PTGS2 is the key enzyme in prostaglandin biosynthesis [71]. *TNFAIP6* gene coding hyaluronan binding proteins is necessary for cumulus expansion [72]. The earlier study showed that *HAS2* and *PGR* expressions in the CCs of the mice receiving MT increased compared to the control groups [73], and Tian et al. [74] found that the MT has efficient ovine cumulus expansion. Furthermore, it has been demonstrated that FGF2 in CCs plays a crucial role in promoting cumulus expansion and increasing the expression of genes associated with expansion (*HAS2* and *PTGS2*) [75,76]. Yuan et al. [19] have found that stress-related genes (*CYP11A1*, *BAD*, and *TP53*) were down-regulated (*p* < 0.05) in CCs from COCs cultured in an FLI medium. Our experiments showed that the mRNA expression of *HAS2*, *TNFAIP6*, and *PTGS2* was significantly improved by the combination treatment of CNP, MT, and FLI, indicating that the CCs expansion factors were increased (Figure 7). On the contrary, the mRNA expression of *CYP11A1*, *BAD*, and *TP53* was significantly decreased, indicating that the stress of CCs was inhibited by the combined treatment of CNP+MT+FLI.

## 5. Conclusions

The combination of CNP, MT, and FLI during IVM significantly improved the quality and development ability of bovine oocytes. Likewise, a combination of CNP, MT, and FLI also increased the ATP, GSH, TZP, ΔΨm, cacline-AM fluorescence intensity of oocytes, and decreased the ROS of oocytes. Our findings inferred that combining CNP, MT, and FLI in the maturation medium resulted in a profound improvement in oocyte competence and in vitro embryo development.

Elucidating the importance of CNP, MT, and FLI for cytobiochemical and molecular balance of intracellular anti- and pro-oxidative processes in IVM-derived heifer/cow oocytes might be helpful for elaborating a variety of biotechnological and embryological parameters. The combination of these parameters might result in the efficient generation of bovine and other mammalian high-quality embryos not only by standard IVF [77,78] but also by intracytoplasmic sperm injection (ICSI) [79,80] or by somatic cell nuclear transfer (SCNT) [81,82]. 

## Figures and Tables

**Table 1:** Primers used for qRT-PCR of candidate genes in CCs, oocytes, and blastocysts.

	Gene	Primers (5′–3′)	Size (bp)	GenBank Accession No.
CCs	*HAS2*	F: CCTCATCATCCAAAGCCTGT	170	NM_174079
		R: CGGGGTAGGTTAGCCTTTTC		
	*TNFAIP6*	F: CATCTTGCCACCTACAAGCA	225	NM_001007813
		R: CACACCACCACACTCCTTTG		
	*PTGS2*	F: GAAATGATCTACCCGCCTCA	283	NM_174445
		R: GCAGCTCTGGGTCAAACTTC		
	*CYP11A1*	F: GTCCTGAACACGGAGGTGAT	293	NM_176644
		R: ACGTTGAGCAGAGGGACACT		
	*BAD*	F: TCAACCAGGACTGGAGGAAG	119	NM_001035459
		R: GAGGATGAGCGACGAGTTTC		
	*TP53*	F: CCTCTCCACAGCCAAAGAAG	121	NM_174201
		R: AGAGCATCCTTCAGCTCCAA		
oocytes	*MPS1*	F: CCATGGGAACGGAAGAGTTA	202	XM_024996643
		R: TAACCGTCCCAACCTGAGAG		
	*Mad1*	F: ATGGCAGGAAGCTAACCAGA	91	NM_001102044
		R: TTGCTCCAAATCCTTGATCC		
	*Mad2*	F: TGGCCGAGTTCTTCTCATTT	82	NM_001191513
		R: TGCACCCGAGTAAAGGTTTC		
	*Myo10*	F: TCAAGCCAAACATGCAGAAG	296	NM_174394
		R: CTCCAGTTTCTGCTCCAAGG		
	*FScn1*	F: CGCCAGATGCTACTTTGACA	297	NM_001035045
		R: CCCGTGGAGTCTTTGATGTT		
	*Daam1*	F: GCTGTGTCAGAAGCCAAACA	149	NM_001081588
		R: CCGCCTTCTTCACTGTTCTC		
blastocyst	*NDUFS8*	F:AAGCCGCAGTAGATGCACTT	249	NM_001302669
		R:GAGCTACCTGTTCCGTGAGC		
	*TFAM*	F:CCAGTCTGCCCTGTAAGCAT	240	NM_001034016
		R:CGACTGCGCTATCCCTTTAG		
	*PINK1*	F:GTGGCTGCTAATGTGCTTCA	140	NM_001099701
		R:TTCTTCTCCGTCAGCCTGTT		
	*BCL2*	F: CATCGTGGCCTTCTTTGAGT	111	NM_001166486
		R: CGGTTCAGGTACTCGGTCAT		
	*BAX*	F: TCTGACGGCAACTTCAACTG	205	XM_015458140
		R: TGGGTGTCCCAAAGTAGGAG		
	*Β-ACTIN*	F: CTCTTCCAGCCTTCCTTCCT	178	NM_173979
		R: GGGCAGTGATCTCTTTCTGC		

**Table 2:** The combination treatment of CNP, MT, and FLI on the maturation ability of oocytes.

	Groups	No. COCs	No. MII Oocytes
	Control	287	217 (75.61 ± 5.02%) c
	CNP	233	195 (83.69 ± 4.85%) b
	CNP+MT	225	192 (85.33 ± 3.57%) b
	CNP+MT+FLI	241	224 (92.95 ± 2.48%) a
OPU	Control	169	138 (81.66 ± 2.63%) b
OPU	CNP+MT+FLI	147	133 (90.48 ± 3.14%) a

a, b, c, d Values with different superscripts indicate significant differences between groups (*p* < 0.05).

**Table 3:** The combination treatment of CNP, MT, and FLI on the developmental ability of bovine oocytes and IVF-derived.

	Groups	No. Cleavage $break$Embryos	No. Blastocysts	Survival Rate after $break$Vitrification	Hatched Rate
	Control	73.51 ± 2.47% (136/185) ^d^	30.15 ± 2.15% (41/136) ^d^	90.32 ± 8.17% (28/31) ^b^	75.00 ± 6.75% (21/28) ^c^
	CNP	79.58 ± 3.14% (113/142) ^c^	35.40 ± 1.90% (40/113) ^c^	90.00 ± 8.43% (27/30) ^b^	85.19 ± 7.26% (23/27) ^b^
	CNP+MT	85.03 ± 4.84% (125/147) ^ab^	42.40 ± 3.94% (53/125) ^b^	91.43 ± 7.19% (32/35) ^b^	87.50 ± 6.48% (28/32) ^b^
	CNP+MT+FLI	91.61 ± 5.17% (142/155) ^a^	51.41 ± 4.73% (73/142) ^a^	97.37 ± 5.35% (37/38) ^a^	94.59 ± 8.23% (35/37) ^a^
OPU	Control	75.22 ± 4.38% (82/109) ^c^	30.49 ± 1.87% (25/82) ^d^	-	-
OPU	CNP+MT+FLI	89.36 ± 4.25% (84/94) ^a^	41.67 ± 2.17% (35/84) ^b^	-	-

a, b, c, d Values with different superscripts indicate significant differences between groups (*p* < 0.05).

## Data Availability

The data used to support the findings of this study are included within the article.
